# A review of common methods to convert morphine to methadone

**DOI:** 10.3402/jchimp.v2i4.19541

**Published:** 2013-01-07

**Authors:** Eric Wong, Kathryn A. Walker

**Affiliations:** 1 University of Maryland School of Pharmacy, Baltimore, MD, USA; 2 Department of Pharmacy Practice and Science, University of Maryland School of Pharmacy, Baltimore, MD, USA; 3 Department of Medicine, Union Memorial Hospital, Baltimore, MD, USA

**Keywords:** methadone, opioid rotation, conversion, pain management, equianalgesic

## Abstract

When dosed appropriately on carefully chosen patients, methadone can be a very safe and effective choice in managing chronic pain. Many authors have discussed important issues surrounding patient selection, drug interactions, screening for QTc prolongation and monitoring. This article will focus on the dosing dilemma that exists after the patient is deemed an appropriate candidate for methadone and a conversion is necessary from another opioid. Despite many publications dedicated to addressing this challenging topic, there is no consensus on the most appropriate method for converting an opioid regimen to methadone. Given the lack of concrete guidance, clinicians in a community setting are likely to be faced with an increased challenge if there are no available pain specialists to provide clinical support. Common methods for converting morphine to methadone will be reviewed and two clinical patient scenarios used to illustrate the outcomes of applying the methods.

The role of methadone in pain management is often misunderstood as it is traditionally associated with opioid agonist therapy related to substance abuse. However, when dosed appropriately on carefully chosen patients, methadone can be a very safe and effective second-line option in managing chronic pain (1–4).

Utilizing methadone as an analgesic can be complicated by the pharmacokinetic and pharmacodynamic profile (5–8). This presents a barrier when compared with more predicable opioids such as morphine. Many authors have discussed important issues surrounding patient selection, drug interactions, screening for QTc prolongation and monitoring (9–12). This article will not discuss making the decision of whether to use methadone in a specific patient situation, but instead focus on the dosing dilemma that exists after the patient is deemed an appropriate candidate. Despite many publications dedicated to addressing this challenging topic, there is no consensus on the most appropriate method for converting an opioid regimen to methadone (13–17). Given the lack of concrete guidance, clinicians in a community setting are likely to be faced with an increased challenge if pain specialists are not available to provide clinical support.

Two things that are consistent among the different methods are: 1) the use of oral morphine as the standard opioid to use in the conversion; and 2) unlike other opioids, conversion to methadone is not based on a linear model (12). Methadone's unique mechanism of mu receptor agonism paired with *N*-Methyl-d-Aspartate (NMDA) antagonism is thought to be responsible for diminishing the tolerance developed as doses of other opioids are increased. Despite the evidence supporting this non-linear relationship, the decision of which method to use in clinical practice can be daunting (3, 10, 12, 14, 18–21). We will illustrate the well-known methods for converting to methadone using community hospital case examples (Table 1).

**Table 1:** Case examples

Case 1: A 52-year-old Caucasian man with a history of esophageal cancer status post tracheostomy and PEG tube placement is receiving opioids for the management of chronic pain. His current course of pain medication is morphine 4 mg IV every 3 hours scheduled to avoid swallowing tablets and it has been effectively relieving his pain with minimal use of breakthrough opioid doses. The medical team is considering methadone liquid to provide long acting analgesia.
Case 2: A 66-year-old African-American woman with a history of severe diabetic peripheral neuropathy and low back pain. She has benefited from the last increase in her long acting opioids, however she has presented with myoclonus thought to be related to the increase in dose. Opioid rotation to methadone is considered, requiring a conversion from her current regimen, which is equivalent to 1,600 mg oral morphine.

To begin the process of initiating methadone therapy, one must consider many variables. The agent and patient variables must be carefully examined in light of the clinical scenario to determine whether a patient is an appropriate candidate for methadone therapy. Both case example patients have been screened and determined to be appropriate for methadone therapy, however a conversion method must be chosen.

## Methadone dosing methods

The manufacturer of methadone recommends that opioid-naïve patients receive methadone 2.5–5 mg PO every 8–12 hours (20). However, it is common for patients in a community hospital setting who are candidates for methadone therapy to be rotating from another opioid and not opioid-naïve. Commonly cited methods and their corresponding ratios are described below for use in performing methadone conversions for patients currently receiving another opioid.

In 1998, Ripamonti et al. proposed a set of conversion ratios that were stratified based on the initial oral morphine dose (Table 2). Smaller proportions were used for daily oral morphine equivalents over 300 mg. One criticism of this method and other methods that stratify ratios based on ranges of total daily morphine use is the illogical nature of the cut-points (e.g., 88 mg of morphine=22 mg oral methadone, whereas 93 mg of morphine=16 mg oral methadone). At doses near the cut-point between ratios, providers should be cautious in not over-estimating the dose.

**Table 2:** Summary of dosing ratios

Ripamonti (18)						
Morphine dose (mg/day)	30–90		90–300		>300	
Morphine: methadone ratio	4:1		6:1		8:1	
Ayonrinde (3)						
Morphine dose (mg/day)	<100	101–300	301–600	601–800	801–1,000	>1,001
Morphine: methadone ratio	3:1	5:1	10:1	12:1	15:1	20:1
Mercadante (4)						
Morphine dose (mg/day)	30–90		90–300		>300	
Morphine: methadone ratio	4:1		8:1		12:1	
Methadone product information (20)						
Morphine dose (mg/day)	<100	100–300	300–600	600–1,000	>1,000	
Oral methadone as percent of total daily morphine dose	20–30%	10–20%	8–12%	5–10%	<5%	

**Fig. 1:**
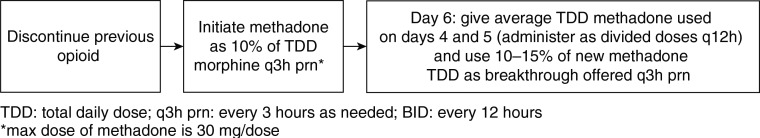
Summary of the Morley–Makin method.

Another proposed model for converting morphine to methadone was presented in 1990 in the form of an algorithm (Fig. 1). The Morley–Makin model directs the clinician to offer a methadone dose equal to 10% of the daily oral morphine dose (but not to exceed 30 mg) as needed every 3 hours (21). After 5 days of administration, the total daily methadone doses required on days 4 and 5 are averaged. This represents the new total daily dose of methadone to be given in divided doses twice daily. In a community hospital setting, this approach can be very resource intensive because of the frequent dose administration required for the first 5 days. If the patient is admitted during this time, it allows for verification of administered doses and increased confidence in calculating a safe dose for maintenance. This method is not ideal to use in a home setting where documentation of each dose and monitoring can be challenging.

Ayonrinde and Bridge also performed a prospective study in 2000 that led to another set of conversion ratios (Table 2) (3). This protocol also calls for a loading dose of 25–50% of the total daily methadone dose to be given on each of the first 2 days.

Another approach published by Mercadante et al. included a set of ratios using the same categories of morphine dose ranges as Ripamonti et al. (Table 2) (19). However, the ratios are different for the categories and they range from 4:1 to 12:1 for doses over 300 mg daily morphine. These ratios represent a more conservative approach compared to Ripamonti et al. except for doses between 30 and 90 mg oral morphine daily where the ratio is the same.

The product information for methadone includes a conversion method also (Table 2) (20). The manufacturer chose to present their conversions in terms of percentages rather than ratios. This method is very similar to Ayonrinde's in terms of proportion and cut-points.

**Fig. 2:**
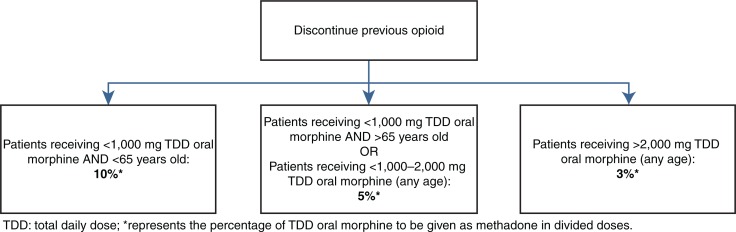
Summary of the Friedman method.

Friedman et al. proposed another algorithm that takes into account the dose of morphine but is unique in also incorporating the patient's age (Fig. 2) (10). This algorithm may underestimate the methadone requirement at lower doses because a very conservative percentage is used for a wide range of possible dosing scenarios. Any calculated methadone dose below that of the manufacturer's stated dose for an opioid naïve patient should be rounded up to a starting dose. Many clinicians prefer using this dosing method because the simplicity makes it easy to memorize and it represents a conservative approach (12).

## Case review

Case 1: As discussed in this review, we must first calculate his total daily oral morphine requirements. At his current dose, he is receiving 32 mg IV morphine daily that is the equivalent of 96 mg oral morphine daily. The summary of applying each method in converting his morphine to methadone reveals a variety of possible courses of action for this patient (Table 3).

**Table 3:** Case 1 example

Method	Ripamonti	Ayonrinde	Mercadante	Product information	Morley–Makin	Friedman
Morphine: methadone ratio	6:1	3:1	8:1	20–30%	10% q3h prn	10%
Methadone daily dose	16 mg	32 mg	12 mg	19–29 mg	10 mg q3h prn*	10 mg

* This is the initial dose offered for days 1–5, not the final calculated dose for day 6.

Based on the methods reviewed, the possible dosages ranged from 10 to 80 mg per day if the patient took every dose offered in the Morley–Makin model. To determine the most appropriate dose, a practitioner must weigh up the risks and benefits of aggressive versus conservative methadone dosing. The Morley–Makin method would not be the most appropriate because this patient is approaching discharge from the hospital. Four of the methods recommend doses between 10 and 20 mg daily, so it would be appropriate to start with 5 mg methadone PO every 8 hours.

**Fig. 3:**
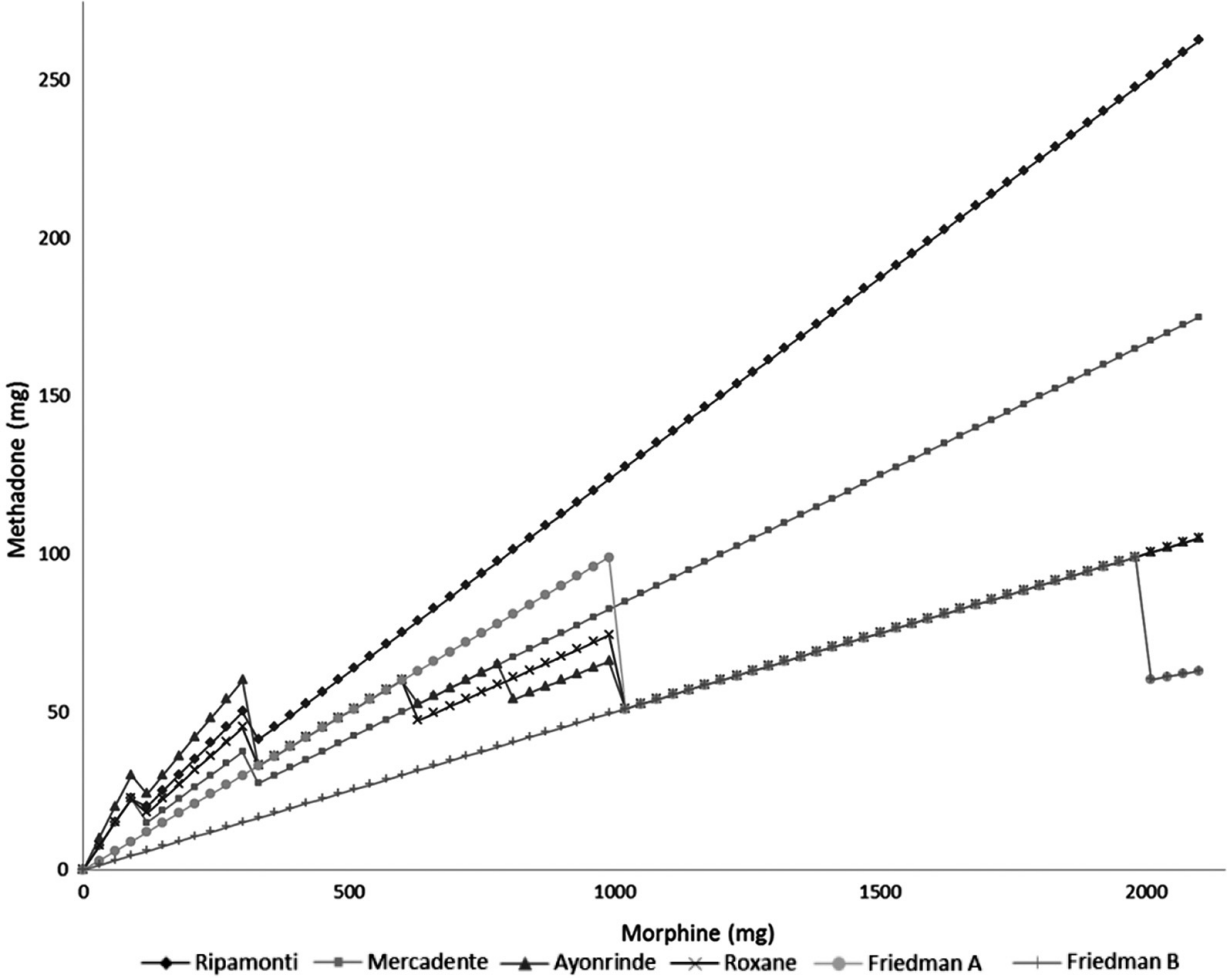
Comparison of methadone dosing methods. Friedman A represents conversions for patient <65 years of age and Friedman B represents conversions for patient >65 years of age.

The convenience of converging doses with different methods is lost at higher doses. Figure 3 depicts the degree of divergence in methadone doses as the daily dose of oral morphine equivalents increases. The X-axis represents increasing total daily doses of morphine and the Y-axis represents the corresponding amount of methadone required by each method to provide equianalgesia. The large difference in recommended doses at higher morphine doses reflects the lack of data. The studies on which many of the conversion methods are based rarely included data for doses greater than 1,000 mg morphine/day, however the dosing schemes are left open-ended to include higher doses.

Case 2: Due to the high morphine requirements in this case, this patient developed signs of opioid-induced neurotoxicity and is planned to undergo opioid rotation to methadone from her current dose of 1,600 mg oral morphine equivalents. The summary of applying each method in converting her morphine to methadone reveals a variety of possible courses of action for this patient (Table 4).

**Table 4:** Case 2 example

Method	Ripamonti	Ayonrinde	Mercadante	Product information	Morley–Makin	Friedman
Morphine: methadone ratio	8:1	20:1	12:1	5%	10% q3h prn	5%
Methadone daily dose	200 mg	80 mg	133 mg	80 mg	30 mg q3h prn*	80 mg

* This is the initial dose offered for days 1–5, not the final calculated dose for day 6 (maximum dose recommended in this method).

In examining the existing options for Case 2, the divergence in doses becomes clearer and the decision-making difficulties more apparent. The patient in Case 2 could receive a methadone dose ranging from 80 mg daily using the Friedman method to 240 mg daily if every dose were taken using the Morley–Makin model. For patients on higher dosages of morphine, the lack of evidence forces clinicians to dose very conservatively. Providers that are inexperienced in initiating methadone should seek guidance from someone experienced in dosing methadone before embarking on *any* conversion, but especially one involving high doses. The most conservative dose produced from the various methods would be recommended and starting even lower would be prudent. Therefore, using the 20:1 conversion (i.e., 5%=80 mg) would represent the highest dose recommended in this situation. Starting with 20 mg methadone given every 8 hours would be reasonable, along with providing liberal breakthrough doses of short acting opioids to cover uncontrolled pain.

## Discussion

It is often difficult to determine the clearest path in transitioning patients to methadone from other opioids in light of the variety of methods available, which sometimes conflict. However, a clinician motivated to use methadone should remember that opioid rotation should not be focused on the exact numerical dose of the method, but on the importance of individualized titration, diligent assessment and careful monitoring (22). At lower doses of morphine, most of the methods reviewed are reasonable and result in comparable doses. The Friedman method does appear to produce the most conservative conversion across all dosage ranges. In contrast, the Ayonrinde method is the most aggressive at lower doses (i.e., <300 mg morphine/day), in line with other methods in moderate doses (i.e., 300–1,000 mg morphine/day) and equally as conservative as the Friedman method at doses over 1,000 mg morphine/day. The Friedman method is the only method that encourages clinicians to adjust the ratio after the threshold of 2,000 mg morphine/day. Doses above this level should be approached with utmost caution and much guidance from experienced pain specialists.

In addition to the six methods reviewed, there are other methods published in the various resources. This article was not meant to review every possibility of methadone conversion, but to focus on the most commonly used methods. A systematic review of the evidence supporting methadone conversions concluded that no one method is superior to another (22). Until new data emerges to allow a true equianalgesic relationship to be determined, clinical judgment and a review of timely and relevant methods should be carefully applied and individualized to care for patient pain effectively and safely.
